# Radiological Outcomes and Complications of Vertical Expandable Titanium Rib Instrumentation in Congenital Scoliosis With or Without Rib Fusion: A Retrospective Study

**DOI:** 10.7759/cureus.14167

**Published:** 2021-03-29

**Authors:** Ozair Bin Majid, Zayed S Al-Zayed, Abdullah M Alsultan, Ali Altalhy, Nouf F Alsadoun, Omar A Al-Mohrej

**Affiliations:** 1 Department of Orthopaedic Surgery, King Faisal Specialist Hospital and Research Centre, Riyadh, SAU; 2 Division of Orthopaedic Surgery, Department of Surgery, McMaster University, Hamilton, CAN

**Keywords:** surgical complications, vertical expandable titanium rib, instrumentation, congenital scoliosis, rib fusion, thoracic, spine

## Abstract

Introduction: In congenital anomalies of the thoracic spine, fusion in situ and hemi-epiphysiodesis are unsuitable surgical options, because three-dimensional thoracic deformity and insufficiency are uncorrectable. We aimed to evaluate the radiological outcome of vertical expandable titanium rib (VEPTR) application after follow-up in children with congenital scoliosis with or without rib fusion.

Methods: In our study, we included 58 patients with congenital scoliosis with or without fused ribs; all treated with VEPTR from 2005 to 2015 at our institute. There were 19 males and 39 females. For each patient, we collected information about age at the index surgery (VEPTR application) and the total number of VEPTR lengthening procedures. Also, Cobb angle, kyphotic angle, thoracic height, and spinal height were measured on preoperative radiographs, immediately post-operative, two years post-operative, and at final follow-up.

Results: The mean duration of follow-up was five years (range, 2-12 years). Twenty-eight patients had rib-to-pelvis type VEPTR, 20 patients had rib-to-rib type VEPTR, and 10 patients had a rib to pedicle/lamina type of VEPTR implant. Post-VEPTR, 63.8% of our patients reported one or more complications. The immediate post-VEPTR application showed that the mean Cobb angle decreased to 43.56° with a percentage change of 22.8% (p<0.001). The mean increase in thoracic height between VEPTR application surgery and final follow-up was 32 mm with a 19.3% increase (p<0.001). Similarly, the mean increase in the spinal height between the VEPTR application surgery and final follow-up was 46.6 mm, with a 23% increase (p<0.001).

Conclusions: VEPTR instrumentation for congenital scoliosis, with or without rib fusion, successfully corrects the coronal Cobb angle in the majority of patients. It also allows the thoracic (T1-T12) and spinal (T1-S1) growth to approach normal for a particular age.

## Introduction

Congenital scoliosis represents an abnormal spinal curvature caused by failure of vertebral formation and/or segmentation associated with fused ribs [1]. In congenital anomalies of the thoracic spine, fusion in situ and hemi-epiphysiodesis are unsuitable surgical options, because three-dimensional thoracic deformity and insufficiency are uncorrectable [2]. Vertical expandable titanium rib (VEPTR) implantation may be considered to treat thoracic insufficiency syndrome (TIS) associated with congenital scoliosis, Jeune syndrome, and Jarcho-Levin syndrome [3]. The VEPTR was established by Campbell et al. during the 1990s to manage TIS; they challenged the notion that guided or forced growth management of congenital scoliosis deformities which could not be done in congenital abnormalities with the use of rib-based systems using the VEPTR [1,4]. The aim of the VEPTR application is to increase the capacity for lung development by constricted chest wall elongation. As a result, progression of scoliosis is preventable, and thoracic growth is allowed [2].

In congenital scoliosis with or without rib fusions, application of rib-to-rib or rib-to-spine-based distraction devices will elongate the concave side by an average of 8 mm/year. On the other hand, the convex side of the deformity will be elongated by 8.3 mm/year over a mean follow-up period of four years [4]. A variety of implants, such as rib-to-rib, a hybrid that is a rib to laminar hook or pedicle screw, or a rib-to-pelvis implant, can be used [5].

We aimed to evaluate the radiological outcome of VEPTR application after follow-up in children with congenital scoliosis with or without rib fusion as we think that VEPTR application with or without expansion thoracoplasty in congenital scoliosis may prevent spinal deformity from progressing, in addition to allowing spinal and lung growth.

## Materials and methods

In our study, we initially included 75 patients; all treated with VEPTR from 2005 to 2015 at our institute. We excluded patients with spina bifida and other syndromic deformities. Patients with any spinal surgery before the index VEPTR procedure were also excluded; and we finally focused on 58 patients with congenital scoliosis with or without fused ribs.

There were 19 males and 39 females. For each patient, we collected information about age at the index surgery (VEPTR application) and the total number of VEPTR lengthening procedures. Also, Cobb angle, kyphotic angle, thoracic height, and spinal height were measured on preoperative radiographs, immediately post-operative, two years post-operative, and at final follow-up. Patients were also evaluated for the development of minor and/or major complications.

Preoperative evaluation

Patient selection for surgery was performed after evaluation by a multidisciplinary team, including a pediatric orthopedic surgeon, a pediatric pulmonologist, and pediatric anesthesia consultation. Radiological evaluation of the deformity was performed with plane radiography and CT scans. MRI of the whole spine was also performed for the patient, to evaluate neural axis abnormalities such as diastematomyelia, tethered cord, and syrinx. Surgical intervention for intrathecal anomalies was undertaken, if required, before correction of the spinal deformity. Ideally, preoperative evaluation should also include pulmonary function tests to evaluate lung function. Unfortunately, due to the unavailability of pulmonary function tests in infants and very young children, it was not included in our study.

Surgical technique

The surgical incision in the posterior wall is the access for VEPTR instrumentation, and the same incision is used for lengthening. The selected level for the proximal cradle should be well within the cephalad part instead of proximal to the curve. The inferior implant could be a distal rib cradle, pedicle screw, laminar hook, or alar hook. The closure was performed keeping an adequate muscle flap over the prominent portion of the VEPTR. The skin was closed with absorbable monofilament sutures. Six weeks postoperatively, patients returned to their activities, and a brace was not required.

VEPTR lengthening

At intervals of an average of six months, the device was expanded during elective surgery. To prevent acute fixation loosening and rib fracture, the device was elongated slowly to reach a desirable amount of reactive force [4].

Radiographic measurements

We measured the Cobb angle, thoracic kyphosis, thoracic height of T1 to T12, and spinal height of T1 to S1, on plain thoraco-lumbar radiographs. The T1-T12 height was measured between the midpoints of the T1 upper endplate and the T12 lower endplate. Similarly, T1 to S1 spinal height was measured between the midpoints of the T1 upper endplate and S1 upper endplate. Hospital records were reviewed for types of surgical procedures, including the number of lengthening procedures and complications.

Statistical analysis

The data were analyzed using the Statistical Product and Service Solutions (SPSS. IBM Corp. Released 2020. IBM SPSS Statistics for Windows, Version 27.0. Armonk, NY: IBM Corp). Continuous data were presented as mean ± standard deviation, and categorical data were presented as frequency (% from total). Spine and thoracic heights pre-VEPTR and post-VEPTR were compared with average normal spine and thoracic heights age-wise, and the improvements were reported as percentage differences. P-values calculated for differences between the genders by chi-square test; independent sample t-test and Mann-Whitney U-test for categorical, normal, and non-normal variables, respectively. P<0.05 is considered a significant difference.

## Results

Our study comprised 58 subjects: 39 females and 19 males. At the time of the VEPTR application, 23 patients were 1-4 years of age (39.7%), 21 were 5-7 years old (36.2%), 6 patients were 8-10 years old, and eight patients were 11-14 years old. The mean age at the time of VEPTR application was 5.95 years (range, 1 year 3 months to 14 years). The average number of expansion procedures was 4.26 (range, 1-9). The mean duration of follow-up was five years (range, 2-12 years). Twenty-eight patients had rib-to-pelvis type VEPTR, 20 patients had rib-to-rib type VEPTR, and 10 patients had a rib to pedicle/lamina type of VEPTR implant (Table 1). Thoracotomy was performed for patients with fused ribs. Post-VEPTR, 63.8% of our patients reported one or more complications.

**Table 1:** Baseline characteristics of the patients. Data are shown as N (%) for categorical variables (#); and mean ± SD and range for normal continuous variables (^). VEPTR: vertical expandable titanium rib, SD: standard deviation.

Type of VEPTR		
Rib-to-pelvis	28 (48.3)	
Rib-to-rib	20 (34.5)	
Rib to pedicle/lamina	10 (17.2)	
	Mean ± SD	Range
Age (years)^#^	5.95 ± 3.2	1-14
Number of total expansions^#^	4.26 ± 2.0	1-9
Baseline to final follow-up years^#^	5.78 ± 2.3	2-12
Cobb angle (°) at baseline^	56.42 ± 19	25.8-125.3
Thoracic height (mm) at baseline^	142.31 ± 28.8	94-212
Spine height (mm) at baseline^	234.02 ± 48	149.6-370
Kyphosis angle (°) at baseline^	40.11 ± 14.1	14.3-80

Prior to implantation, the mean primary Cobb angle was 56.42° ± 19° (range, 25° to 125°) and mean thoracic kyphosis was 40.11° ± 14.1° (range, 14° to 80°). The immediate post-VEPTR application showed that the mean Cobb angle decreased to 43.56° with a percentage change of 22.8% (p<0.001). However, this change in the Cobb angle decreased with time, with a final average Cobb angle reaching 55.41° (percentage change of 1.8%). It was observed that the thoracic kyphosis angle progressively increased over time, and at the final follow-up, an average increase of 6° (15.6%) was seen compared to pre-VEPTR values. The mean increase in thoracic height between VEPTR application surgery and final follow-up was 32 mm with a 19.3% increase (p<0.001). Similarly, the mean increase in the spinal height between the VEPTR application surgery and final follow-up was 46.6 mm, with a 23% increase (p<0.001) (Table 2).

**Table 2:** Change after VEPTR treatment compared to pre-VEPTR. Data are shown as mean ± SD. Change post-VEPTR treatment and follow-ups are shown as difference at follow-up minus baseline (% change at follow-up). Differences between the follow-up stage (post-VEPTR, at 2 years post-VEPTR and final follow-up) compared to the pre-VEPTR were calculated by paired samples t-test. VEPTR: vertical expandable titanium rib, SD: standard deviation.

VEPTR	Average ± SD	Change from pre-VEPTR	P-value
Cobb’s angle (°)			
Pre-VEPTR	56.42 ± 19	-	-
Post-VEPTR	43.56 ± 15.8	−12.9 (−22.8)	<0.001
At two-year follow-up	51.51 ± 17.5	−4.9 (−8.7)	0.15
Final follow-up	55.41 ± 17.3	−1 (−1.8)	0.76
Kyphosis angle (°)			
Pre-VEPTR	40.11 ± 14.1	-	-
Post-VEPTR	41.3 ± 12.3	1.2 (3)	0.63
At two-year follow-up	43.05 ± 14.3	2.9 (7.3)	0.27
Final follow-up	46.38 ± 16.7	6.3 (15.6)	0.03
Spine height (mm)			
Pre-VEPTR	234.02 ± 48	-	-
Post-VEPTR	252.11 ± 46.6	18.1 (7.7)	0.04
At two-year follow-up	260.38 ± 50	26.4 (11.3)	0.005
Final follow-up	279.13 ± 56.4	45.1 (19.3)	<0.001
Thoracic height (mm)			
Pre-VEPTR	142.31 ± 28.8	-	-
Post-VEPTR	152.24 ± 28.6	9.9 (7)	0.06
At two-year follow-up	157.98 ± 29.7	15.7 (11)	0.005
Final follow-up	175.08 ± 38.9	32.8 (23)	<0.001

**Figure 1:**
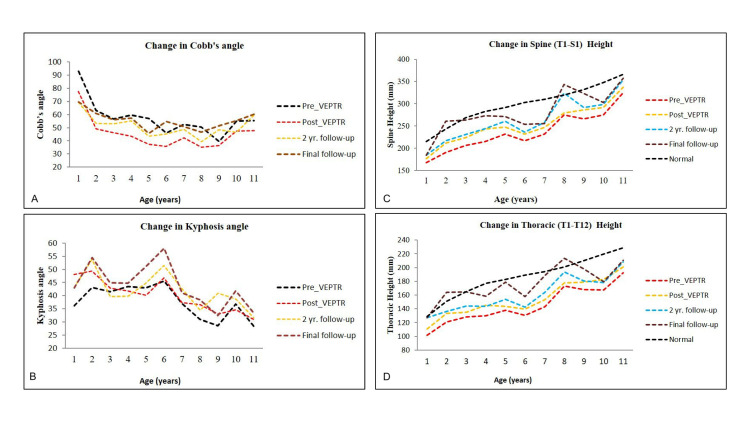
Comparison of spine height and thoracic height after VEPTR application. (A) Change in Cobb angle before and after VEPTR treatment. The data points for all subjects were calculated using the average Cobb angle. (B) Change in thoracic kyphosis before and after VEPTR treatment. The data points for all subjects were calculated using the average kyphosis angle. (C) Change in spinal height (T1–S1) at different stages of the VEPTR treatment, compared with normal thoracic height as a function of age. (D) Change in thoracic height (T1–T12) at different stages of the VEPTR treatment, compared with normal thoracic height, as a function of age.

The spine height and thoracic height of the patients after VEPTR application were compared with age-wise normal spine and thoracic spine heights (Figure 1).

Before VEPTR application, the average spine and thoracic heights were 22.4% and 15.2% lower than the normal. Immediately after VEPTR application, the difference was reduced to 16.4% and 11.9%, respectively, for spine and thoracic heights compared to normal. After two years post-VEPTR application, the average difference compared to normal still reduced to 13.6% and 10.1%, respectively, for spine and thoracic heights while at final follow-up, compared to normal spine and thoracic heights, the difference reduced to 7.4% and 4.3%, respectively. The results are presented in Table 3.

**Table 3:** Improvement in spinal height and thoracic height post-VEPTR treatment compared to an age-wise normal spine. Data are shown as mean ± SD. Improvement post-VEPTR treatment is shown as average differences compared to age-wise normal values (% change). VEPTR: vertical expandable titanium rib, SD: standard deviation.

VEPTR	Average ± SD	Change with respect to normal
Spine height (mm)		
Normal as per age	301.43 ± 41.8	-
Pre-VEPTR	234.02 ± 48	67.4 (22.4)
Post-VEPTR	252.11 ± 46.6	49.3 (16.4)
At two-year follow-up	260.38 ± 50	41.1 (13.6)
Final follow-up	279.13 ± 56.4	22.3 (7.4)
Thoracic height (mm)		
Normal as per age	188.14 ± 27.1	-
Pre-VEPTR	142.31 ± 28.8	45.8 (15.2)
Post-VEPTR	152.24 ± 28.6	35.9 (11.9)
At two-year follow-up	157.98 ± 29.7	30.2 (10.1)
Final follow-up	175.08 ± 38.9	13.1 (4.3)

**Figure 2:**
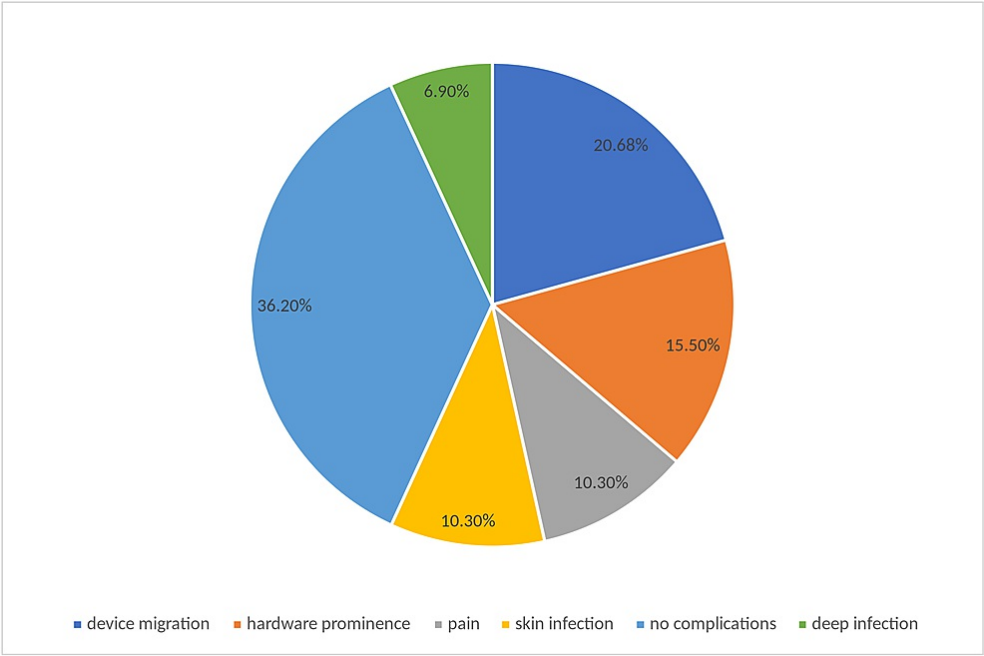
Complications of VEPTER found within our study.

Unfortunately, VEPTR instrumentation has many potential complications. Implant migration was seen in 12 patients; severe hardware prominence was seen in 9 (15.5%) patients; skin infection and pain were reported in 6 (10.3%) patients; and deep infection was noted in four of our patients. Complications after VEPTR instrumentation are presented in Figure 2.

## Discussion

Our study evaluated the radiological outcome of VEPTR application over a mean of five years of follow-up in children with congenital scoliosis with or without rib fusion. We revealed that VEPTR application for congenital scoliosis resulted in moderate correction of the Cobb angle over time. It was observed that maximum correction in the Cobb angle was achieved in the immediate post-surgical phase, and this correction decreased in future follow-ups. We observed an increase in kyphosis over time. Complications were also observed. Anchor migration, implant failure, wound infections, and loss of control of the deformity have all been reported [6-8].

Flynn et al., in their study of 24 patients followed over an average of 40.7 months after VEPTR application, reported an average improvement of 8.9° in Cobb angle [2]. All patients had an increase in thoracic height, with a mean increase of 3.41 cm. They also reported device migration and skin infection as complications [2]. Emans et al. reported an increase in chest volume on post-operative CT scans [8]. Some studies have reported improvement in pulmonary function after VEPTR application in conventional pulmonary function testing. However, children who undergo VEPTR treatment for early-onset scoliosis are not mature enough, or mentally and physically competent, to co-operate with such studies [9]. Dede et al. report improvement in the coronal Cobb angle at final follow-up, but observed an increase in proximal thoracic kyphosis [9]. Patients had a mean gain in T1-T12 height of 18 mm. In a study by Motoyama et al. [10], involving ten patients before the last expansion thoracoplasty, the Cobb angle was measured and compared to the preoperative value and showed an average improvement of 14.4°. Campbell and Hell, in their recent study of 23 children with an average starting age of 3.3 years, have documented significant growth of the spine during VEPTR and expansion thoracostomy [1].

Emans et al. report an increase in mean thoracic spine length by 2.0 mm at the time of initial surgical intervention [8]. Furthermore, in 30 of 31 patients, control of progressive spinal deformity was achieved, with an improvement of Cobb angle to 39° after surgery, compared to 55° before surgery, which is comparable to our observation [8]. Karol et al. have shown that shortening of the T1-T2 index and decreasing AP diameter are due to early arthrodesis [11]. Respiratory insufficiency, in addition to spinal deformity, is caused by fusion. The forced vital capacity may decrease to less than 50% of the predicted volume if more than 60% of the thoracic spine (eight thoracic volumes) are fused before the age of eight years [12,13].

Dimeglio et al. have extensively studied spine growth at different ages and the effect of spinal deformity on the growing spine [6]. In our study, we observed that both the thoracic height (T1-T12) and spinal height (T1-S1) were significantly diminished in the pre-VEPTR period. We noticed a significant improvement in both thoracic height and spinal height over the treatment period, in such a way that the thoracic height and spinal height in the final follow-up phase approached the normal spinal and thoracic growth.

Complications are common in VEPTR treatment, including migration of the superior cradle, migration of the pelvic hooks, and skin and soft tissue infections [14-16]. Complications have been observed to increase with the number of surgeries [17-20]. Lattig et al. [21], in a radiographic evaluation of five children with early-onset spinal deformity, noted the development of auto-fusion in the spine. Moreover, the migration of laminar hooks and rib cradles causes bony bridges between the laminate and ribs. In their study of 63 patients followed over a mean of 2.2 years, El-Hawary et al. noticed a decrease in Cobb angle by 47°, followed by a slight increase at two-year follow-up [22]. Kyphosis also showed a significant decrease after implantation; however, it increased after two years. They further concluded that the spine continues to grow after VEPTR instrumentation [22]; and reported that 31 patients (49%) had at least one complication, with a total of 58 complications. Ramirez et al. reported a complication rate of 13%, including infection, rib fracture, and device migration [23]. We encountered implant migration in 12 patients, which was revised in the subsequent lengthening procedure; severe hardware prominence was observed in nine (15.5%) patients, and skin infection and pain were reported in six (10.3%) patients. We also observed deep infection in four of our patients, two of which were treated with irrigation, debridement, and IV antibiotics, without the removal of the implant. The implant was removed prematurely in the remaining two cases.

A limitation of this study is that the sample cohort is relatively small; nevertheless, the findings showed great promise. While every patient who underwent VEPTR application was included, retrospective studies are open to many types of bias that could affect the results. Although we selected a sample cohort with extensive follow-up data, the study was undertaken in just one center, which affects the results' generalizability. So, our recommendations should be taken into consideration with the limitations and consider similar studies in the literature. Further studies taking into consideration these limitations and involving control groups would be of great value.

## Conclusions

Early-onset congenital scoliosis is certainly a difficult condition to treat. One of the options in treating such conditions is VEPTR instrumentation. In our article, VEPTR instrumentation for congenital scoliosis, with or without rib fusion, successfully corrects the coronal Cobb angle in the majority of patients. It also allows the thoracic (T1-T12) and spinal (T1-S1) growth to approach normal for a particular age. The challenges of VEPTR instrumentation are the different complications associated with it, such as hardware prominence, device migration, infection (superficial and deep), and pain. However, the findings of the present study may be of particular interest to pediatric spine surgeons, as the associated high rates of complications would have effects on the final outcomes.

## References

[1] Campbell RM Jr, Hell-Vocke AK (2003). Growth of the thoracic spine in congenital scoliosis after expansion thoracoplasty. J Bone Joint Surg Am.

[2] Flynn JM, Emans JB, Smith JT, Betz RR, Deeney VF, Patel NM, Campbell RM (2013). VEPTR to treat nonsyndromic congenital scoliosis: a multicenter, mid-term follow-up study. J Pediatr Orthop.

[3] Campbell RM Jr, Smith MD, Mayes TC (2003). The characteristics of thoracic insufficiency syndrome associated with fused ribs and congenital scoliosis. J Bone Joint Surg Am.

[4] Campbell RM, Smith MD, Hell-Vocke AK (2004). Expansion thoracoplasty: the surgical technique of opening-wedge thoracostomy. Surgical technique. J Bone Joint Surg Am.

[5] Hell AK, Campbell RM, Hefti F (2005). The vertical expandable prosthetic titanium rib implant for the treatment of thoracic insufficiency syndrome associated with congenital and neuromuscular scoliosis in young children. J Pediatr Orthop B.

[6] Dimeglio A, Bonnel F, Canavese F (2011). Normal growth of the spine and thorax. The Growing Spine: Management of Spinal Disorders in Young Children.

[7] Campbell RM Jr, Smith MD, Mayes TC (2004). The effect of opening wedge thoracostomy on thoracic insufficiency syndrome associated with fused ribs and congenital scoliosis. J Bone Joint Surg Am.

[8] Emans JB, Caubet JF, Ordonez CL, Lee EY, Ciarlo M (2005). The treatment of spine and chest wall deformities with fused ribs by expansion thoracostomy and insertion of vertical expandable prosthetic titanium rib: growth of thoracic spine and improvement of lung volumes. Spine (Phila Pa 1976).

[9] Dede O, Motoyama EK, Yang CI, Mutich RL, Walczak SA, Bowles AJ, Deeney VF (2014). Pulmonary and radiographic outcomes of veptr (vertical expandable prosthetic titanium rib) treatment in early-onset scoliosis. J Bone Joint Surg Am.

[10] Motoyama EK, Deeney VF, Fine GF, Yang CI, Mutich RL, Walczak SA, Moreland MS (2006). Effects on lung function of multiple expansion thoracoplasty in children with thoracic insufficiency syndrome: a longitudinal study. Spine (Phila Pa 1976).

[11] Karol LA, Johnston C, Mladenov K, Schochet P, Walters P, Browne RH (2008). Pulmonary function following early thoracic fusion in non-neuromuscular scoliosis. J Bone Joint Surg Am.

[12] Ramirez N, Flynn JM, Emans JB (2010). Vertical expandable prosthetic titanium rib as treatment of thoracic insufficiency syndrome in spondylocostal dysplasia. J Pediatr Orthop.

[13] Waldhausen JH, Redding GJ, Song KM (2007). Vertical expandable prosthetic titanium rib for thoracic insufficiency syndrome: a new method to treat an old problem. J Pediatr Surg.

[14] Smith JR, Samdani AF, Pahys J, Ranade A, Asghar J, Cahill P, Betz RR (2009). The role of bracing, casting, and vertical expandable prosthetic titanium rib for the treatment of infantile idiopathic scoliosis: a single-institution experience with 31 consecutive patients. Clinical article. J Neurosurg Spine.

[15] Samdani AF, Ranade A, Dolch HJ, Williams R, St Hilaire T, Cahill P, Betz RR (2009). Bilateral use of the vertical expandable prosthetic titanium rib attached to the pelvis: a novel treatment for scoliosis in the growing spine. J Neurosurg Spine.

[16] Schulz JF, Smith J, Cahill PJ, Fine A, Samdani AF (2010). The role of the vertical expandable titanium rib in the treatment of infantile idiopathic scoliosis: early results from a single institution. J Pediatr Orthop.

[17] Akbarnia BA, Emans JB (2010). Complications of growth-sparing surgery in early onset scoliosis. Spine (Phila Pa 1976).

[18] Sankar WN, Acevedo DC, Skaggs DL (2010). Comparison of complications among growing spinal implants. Spine (Phila Pa 1976).

[19] Latalski M, Fatyga M, Gregosiewicz A (2011). Problems and complications in VEPTR-based treatment. Ortop Traumatol Rehabil.

[20] Al-Mohrej OA, Aldakhil SS, Al-Rabiah MA, Al-Rabiah AM (2020). Surgical treatment of adolescent idiopathic scoliosis: complications. Ann Med Surg (Lond).

[21] Lattig F, Taurman R, Hell AK (2016). Treatment of early-onset spinal deformity (EOSD) with VEPTR: a challenge for the final correction spondylodesis: a case series. Clin Spine Surg.

[22] El-Hawary R, Kadhim M, Vitale M, Smith J, Samdani A, Flynn JM (2017). Veptr implantation to treat children with early-onset scoliosis without rib abnormalities: early results from a prospective multicenter study. J Pediatr Orthop.

[23] Ramirez N, Flynn JM, Serrano JA, Carlo S, Cornier AS (2009). The vertical expandable prosthetic titanium rib in the treatment of spinal deformity due to progressive early onset scoliosis. J Pediatr Orthop B.

